# The complete mitochondrial genome of the *Gymnocypris scleracanthus* (Cypriniformes: Cyprinidae)

**DOI:** 10.1080/23802359.2017.1375872

**Published:** 2017-09-09

**Authors:** Chi Zhang, Jing Liu, Wangliang Wang, Jianshe Zhou, Yingzi Pan, Zhenbo Mou

**Affiliations:** a Fisheries Research Institute, Tibet Academy of Agricultural and Animal Husbandry Sciences, Lhasa, Tibet, People’s Republic of China;; b The Fifth Department of Internal Medicine, The People’s Hospital of Tibet Autonomous Region, Lhasa, Tibet, People’s Republic of China

**Keywords:** *Gymnocypris scleracanthus*, mitochondrial genome, phylogenetic

## Abstract

The *Gymnocypris scleracanthus*, a species of genus *Gymnocypris* belongs to Schizothoracinaein Cyprinidae. In this study, the complete mitochondrial DNA genome sequence of *G*. *scleracanthus* was determined. The 16,771 bp long circular molecule contains 13 protein-coding genes, two rRNA genes, 22 tRNA genes, and two non-coding regions, showing a typical vertebrate pattern. These genes except *ND6* and eight *tRNA* genes were encoded on the H-strand. Phylogenetic analysis showed that the *G. scleracanthus* was a part of genus *Gymnocypris* and had closer relationship with *Gymnocypris dobula* and was independent from other species of Psilorhynchus and Labeoninae in Cyprinidae.

The schizothoracinae fishes (Teleostei: Cyprinidae) are widely distributed in a great number of rivers and lakes on the Qinghai-Tibet Plateau, and are important members of the ichthyofauna in the Qinghai-Tibet Plateau and its peripheral regions (Chen and Cao 2000). *Gymnocypris scleracanthus* is an endemic species to China which is distributed in the Lang Tso in Tibet (Wu and Wu 1992). The unique geography and climate characteristics of Qinghai-Tibet Plateau provide us to explore the phylogenetic relationships among the fish species (He and Chen 2006). Till now, there was little information about the complete mitochondrial genomes of *G. scleracanthus* in GenBank. In our study, the complete mitogenome of *G. scleracanthus* was determined, which would be useful for further genetic studies, phylogenetic analysis, and conservation of this species.

The sample of *G. scleracanthus* was obtained from Lang Tso (29°12′33.45″N 87°23′5.46″E) at an altitude of 4228 m in Tibet, China. A small portion of right pelvic fin was excised and preserved in the Fisheries Research Institute, Tibet Academy of Agricultural and Animal Husbandry Sciences. The total genomic DNA was extracted from the pelvic fin preserved in 95% alcohol. The sequence was amplified by PCR with fifteen pairs of primer.

The complete mitochondrial genome of *G. scleracanthus* was 16,771 bp in length (GenBank accession no. KY420179). The mitogenome contains 13 protein-coding genes, 22 tRNA genes, two rRNA genes, and two non-coding regions. The 22 tRNA were scattered among the whole mitochondrion, and they range from 66 (*tRNA^Cys^*) to 76 (*tRNA^Lys^* and*tRNA^Leu^*) in length. Ten AGGGCCCAGGG repeat sequence was between *tRNA^Thr^* and *tRNA^Pro^*. The whole D-loop region was 935 bp in length. The 12S rRNA (960 bp) and 16S rRNA (1682 bp) were located between *tRNA^Phe^* and *tRNA^Leu^* and were separated by *tRNA^Val^*. Among the 13 protein-coding genes, ATP8 (165 bp) took the shortest sequence and ND5 (1824 bp) took the longest, consistent with previous studies (Ji et al. 2013). Besides, 12 genes took the start codon of ATG, while *COX1* got GTG as the start codon. The termination codon of these 13 protein-coding genes had four types, including “TA” in *COX3*, “TAG” in *ND1* and *ATP*8, “T” in five genes (*ND2, ND3, ND6, COX2* and *CYTB*), and “TAA” in five genes (*ND4L*, *ND5*, *ND6, ATP6,* and *COX1*), which was similar with other species in Cyprinidae.

In this study, we reconstructed the phylogenetic tree on the basis of mitochondrial genome data including 16 species of schizothoracine fish and three closely related species (Figure 1). The molecular phylogenetic tree constructed based on the neighbour-joining (NJ) tree using the MEGA5.2 (Gene Codes, Ann Arbor, MI). Phylogenetic analysis showed that the schizothoracine individuals can be divided into two clades, the primordial-grade schizothoracine fishes and the specialized-grade schizothoracine fishes. The *G. scleracanthus* belonged to the specialized grade and had closer relationship with *Gymnocypris dobula* than with other species. The evolutionary relationships of these analyzed species are consistent with previously reported results (He and Chen 2007). The species from the same and adjacent drainages clustered together and had close relationships.

**Figure 1.**
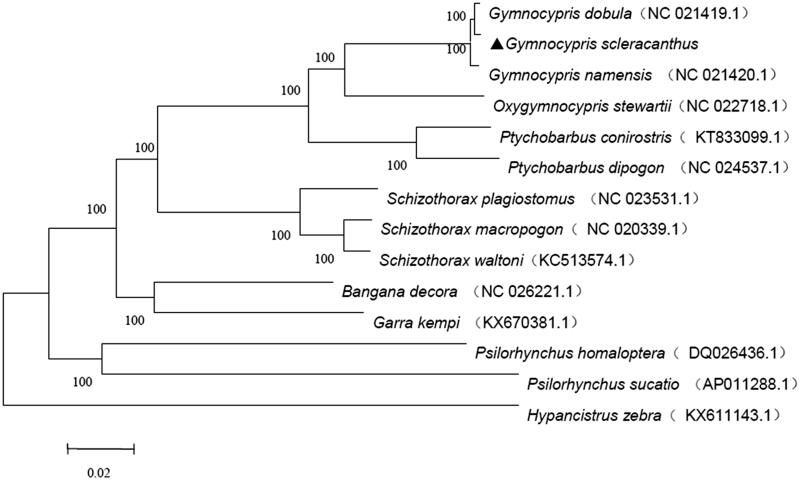
A neighbour-joining (NJ) tree of the *G. scleracanthus* was constructed using mitogenome sequences. The phylogenic tree is constructed by Kimura 2-parameter method with 1000 bootstrap replicates. GenBank accession numbers of mitogenomic sequences for each taxon are shown in parentheses.
